# Kidney Aging and Chronic Kidney Disease

**DOI:** 10.3390/ijms25126585

**Published:** 2024-06-14

**Authors:** Yingying Zhang, Chen Yu, Xiaogang Li

**Affiliations:** 1 Department of Internal Medicine, Mayo Clinic, Rochester, MN 55905, USA; zhang.yingying@mayo.edu; 2 Department of Biochemistry and Molecular Biology, Mayo Clinic, Rochester, MN 55905, USA; 3 Department of Nephrology, Shanghai Tongji Hospital, Tongji University School of Medicine, Shanghai 200092, China; yuchen@tongji.edu.cn

**Keywords:** kidney aging, chronic kidney disease, cell senescence

## Abstract

The process of aging inevitably leads to an increase in age-related comorbidities, including chronic kidney disease (CKD). In many aspects, CKD can be considered a state of accelerated and premature aging. Aging kidney and CKD have numerous common characteristic features, ranging from pathological presentation and clinical manifestation to underlying mechanisms. The shared mechanisms underlying the process of kidney aging and the development of CKD include the increase in cellular senescence, the decrease in autophagy, mitochondrial dysfunction, and the alterations of epigenetic regulation, suggesting the existence of potential therapeutic targets that are applicable to both conditions. In this review, we provide a comprehensive overview of the common characteristics between aging kidney and CKD, encompassing morphological changes, functional alterations, and recent advancements in understanding the underlying mechanisms. Moreover, we discuss potential therapeutic strategies for targeting senescent cells in both the aging process and CKD.

## 1. Introduction

The elderly population has significantly increased in recent years and will account for one-fifth of the population of the globe by 2030 [1]. This imposes an enormous challenge on healthcare systems and has unprecedented health, economic, and social implications worldwide. A generally accepted view is that aging subsumes an organismal phenomenon involving age-related chronic diseases and premature organs with diverse regulatory mechanisms, including genomic instability, telomere attrition, epigenetic alterations, loss of proteostasis, deregulated nutrient sensing, mitochondrial dysfunction, and disabled autophagy [2]. On a cellular level, aging is characterized by cellular senescence, which is an irreversible state of cell cycle arrest and the cessation of cell division [3]. Aging is associated with structural and functional degeneration in major organs of the body, including kidneys.

Chronic kidney disease (CKD) is recognized as a major public health problem affecting >10% of the global population. CKD has been defined by an estimated glomerular filtration rate (eGFR) < 60 mL/min per 1.73 m^2^ over 3 months, which is characterized by progressive and irreversible nephron loss, tubular atrophy, interstitial fibrosis, glomerulosclerosis, vascular rarefaction, and arteriosclerosis [4]. The activation of fibrotic and inflammatory signaling pathways, along with mitochondrial dysfunction, genetic and epigenetic modifications, as well as cellular senescence, have been demonstrated to play pivotal roles in the pathogenesis of CKD [5,6]. Increasing evidence indicates that there is a striking similarity between the manifestations of progressive CKD and aging kidneys [5]. Meanwhile, CKD is often viewed as a form of premature and accelerated aging. Aging kidney and CKD manifest similar changes in their morphology and function, and share many common underlying mechanisms [7].

In this review, we provide a comprehensive overview of the shared characteristics between aging kidney and CKD, encompassing morphological changes, functional alterations, and recent advancements in understanding the underlying mechanisms driving both aging kidney and CKD. Additionally, we discuss potential therapeutic strategies for targeting senescent cells for CKD treatment.

## 2. The Common Structural Changes in Kidneys during Aging and CKD

### 2.1. Anatomical Changes in Kidneys during Aging and CKD

The structure of the kidney undergoes age-related alterations in the process of aging, including a reduction in kidney cortical volume, an increase in surface roughness, and an elevated frequency of renal cyst tumor (often benign) formation at the macrostructural scale. Recent studies show a decline in cortex volume and an increase in medullary volume with age in kidney donors, resulting in the decline in total kidney volume (TKV) until the cortex atrophying [8]. Renal cysts become more frequent with larger size in aged kidneys, especially in males with higher blood pressure and albuminuria patients [9]. The incidence of some benign tumors, such as hamartoma and angiomyolipomas, tends to increase with advancing age. Additionally, the prevalence of parenchymal calcifications, cortical focal scars, fibromuscular dysplasia, and renal artery atherosclerosis also demonstrates a trend to increase in normal aging [9].

In contrast to aging kidney, the structural alterations in the kidneys of patients with CKD develop until the end stages of the disease. The progression of CKD is often accompanied by ongoing renal fibrosis, which is a common feature and prevalent pathological manifestation in all kinds of kidney diseases [10]. When fibrosis progresses to the end stage of CKD, the kidney volume decreases, and the cortex becomes thinner. Simple solitary parenchymal cysts and peripelvic cysts are frequently encountered in CKD patients, especially in those with hemodialysis [11], whereas they generally lack clinical significance [12]. In male patients undergoing long-term hemodialysis, the incidence of acquired cystic disease of the kidney (ACDK) is markedly high. ACDK is known to be accompanied by tumor, bleeding, calculus, abscess, etc. [13]. It has been reported that CKD can also lead to renal cell carcinoma (RCC) [13].

### 2.2. Pathological Changes in Kidneys during Aging and CKD

The pathological changes in aged kidney, resulting from ischemia, hypoxia, and hypertension, include tubular atrophy, inflammation, interstitial fibrosis, glomerulosclerosis, vascular rarefaction, and arteriosclerosis, resembling those observed in CKD [14]. Besides, aged kidney is also characterized by a reduction in the number of nephrons, accompanied by hypertrophy in the remaining nephrons [15].

One of the most frequently observed pathological changes in the aging kidney is an increase in the incidence of focal and global glomerulosclerosis (FGGS) [16]. Various studies indicate that the prevalence of focal and global (but not focal and segmental) glomerulosclerosis escalates with advancing age, even in the absence of evident renal disease [17]. The other changes include decreased number and size of glomeruli, thickening of the glomerular basement membrane, increased mesangial volume and matrix, and wrinkling of capillary tufts [18]. The number of glomerular mesangial cells and endothelial cells increases until age 50, thereby maintaining a physiologically appropriate ratio to the enlarged glomerular volume [19]. However, after the age of 50, both mesangial cells and endothelial cells exhibit a decreasing trend that becomes more pronounced after the age of 70 [19]. Hyaline mesangial matrix expansion leads to the obliteration of the glomerular capillary loops and is associated with capillary tuft collapse and intra-capsular fibrosis. Conversely, a relative decrease in the number of podocytes is observed in the process of aging [20]. In addition, podocytes are experienced with hypertrophy that can be triggered by intracellular absorption of protein droplets, fusion of foot processes, and detachment from the glomerular basement membrane, as evidenced through electron microscopic analysis [21].

Tubular atrophy and interstitial fibrosis are recognized as the predominant tubular-interstitial pathological changes in aging kidney [22]. It is hypothesized that age-related fibrointimal hyperplasia in the small arteries contributes to the development of glomerulosclerosis, subsequently initiating local tubular atrophy and interstitial fibrosis [22]. The kidneys in the aging process exhibit an increased number of glomerular arteries, characterized by direct connections that bypass the afferent and efferent arterioles due to glomeruli loss. The presence of comorbidities, such as hypertension and diabetes, may exacerbate the pathological changes observed in elders.

Vascular abnormalities in aging kidneys are correlated with renal artery atherosclerosis, which can be induced by aging, ischemia, hypertension, obesity, and renal parenchymal hypoxia. Among these alterations, the prevalence of atherosclerosis in renal arteries stands out significantly, starting at 0.4% in young men and escalating to 25% in older individuals [23]. The pathological changes in the glomeruli, blood vessels, and tubulointerstitial lesions associated with aging are not exclusive to aged kidneys but also represent common features leading to renal fibrosis in CKD [24].

## 3. The Common Functional Changes in Kidneys during Aging and CKD

The changes in biological function in aging kidney are characterized by a decline in GFR, impaired tubular function, elevated renal vascular resistance, and endocrine dysfunction. Age-related GFR decline has long been recognized [25], which is an initial parameter used to assess age-related functional decline. Generally, eGFR decreases, beginning at approximately age 30, at a rate of 0.7–0.9 mL/min/1.73 m^2^ per year in healthy individuals. Renal Iohexol Clearance Survey in Troms 6 (RENIS-T6) showed a measured GFR decline rate of 0.95 ± 2.23 mL/min/1.73 m^2^ per year in 1594 healthy individuals (age 50–62 years) [26]. The overall decline in GFR with age, calculated by inulin clearance, starts at the age of 40 and is accelerated at the age of 60–70 [25]. The accumulation of uremic toxins increases in both CKD patients and the elderly. The accumulation of indoxyl sulphate (IS), a byproduct generated through the metabolic breakdown of tryptophan [27], demonstrates an inverse correlation with kidney function, particularly in individuals with eGFR < 60 mL/min [28]. A significant elevation of IS levels was detected among elderly individuals aged 70–89 years, with plasma IS levels of 1.56 ± 0.93 mg/L, which was equivalent to IS levels in CKD stage 3 patients [29]. The increase in serum IS levels has been linked to glomerular sclerosis, heightened oxidative stress, as well as cardiac and renal fibrosis. More importantly, an increase in urea toxins leads to alterations in the intestinal flora that can increase the production of gut-derived toxins and alter the intestinal epithelial barrier, most possibly accelerating the process of CKD [30]. Recently, there has been a growing focus on the influence of gut microbiota in elders. Microbes from old mice, but not young ones, can induce inflammation in germ-free mice, suggesting that microbes become more harmful to the host when they are aged [31]. These studies implicate that gut microbiotadysbiosis is associated with an increase in uremic toxins, thereby accelerating kidney aging and the progression of CKD. The features of age-related tubular dysfunction include a decline in the capacity to concentrate urine, a disruption of electrolyte balance, and an increased susceptibility to AKI [32]. The primary cause of tubular injury is renal interlobular arteriosclerosis, which results in a diminished ability to concentrate or dilute urine. Electrolyte disturbance has been manifested in various ways. There is a reduced urinary sodium excretion among the elderly population, leading to an increased susceptibility to volume overload [33]. In addition, aging negatively affects potassium handling. Urine potassium levels are regulated by active transporters, such as Na-K ATPase, in the tubules and collecting ducts, which play a crucial role in response to decreased GFR and reduced urine output [34].

Age-related renal vascular abnormalities include vascular remodeling, endothelial dysfunction, and an increase in vascular stiffness [35]. The main cause of the elevation in renal microvascular resistance is arteriosclerosis, which is significantly increased with age, especially after the age of 60. Another significant contributing factor to kidney aging is the central role played by the kidney in sympathetic regulation. Due to declining GFR and vascular injuries, elderly individuals exhibit activation of the sympathetic nervous system, leading to accelerated arterial stiffening [36]. The higher incidence of anemia among older adults can be attributed to reduced erythropoietin (EPO) production linked to tubular atrophy or tubulointerstitial scarring [37]. In addition, the levels of hormones associated with the kidneys undergo changes during the process of kidney aging. The serum level of 1,25-dihydroxyvitamin D decreases in the elderly, while the level of 25-hydroxyvitamin D remains normal, suggesting a decrease in conversion rate of 1-dihydroxynitamin D [38], thereby contributing to osteoporosis development among elders [39].

Early changes in renal function among CKD patients are dependent on the underlying cause of the disease. Patients with tubulointerstitial injuries commonly present with anemia, a decreased eGFR, and an electrolyte imbalance, whereas patients with glomerular diseases often manifest with proteinuria. Importantly, in the event that CKD progresses to advanced stages (stage 3b–stage 5, GFR < 45 mL/min), patients will exhibit similar biological dysfunctions as those observed in aged individuals.

Taken together, CKD is often viewed as an accelerated and premature aging of the kidney, while aged kidneys undergo micro- and macro-alterations similar to those observed in CKD. Aging and CKD kidneys share common alterations from structure to function.

## 4. Transplanted Kidney during Aging and CKD

Kidney transplant recipients constitute a distinct cohort, and there is an observed surge in both elderly donors and recipients in light of the current demographic projections and the escalating prevalence of end-stage kidney disease with advancing age [40]. Renal graft deterioration is influenced by various immunological and non-immunological factors, with histological lesions, such as interstitial fibrosis and tubular atrophy, exhibiting similarities to those observed in aging kidneys [41]. It has been proposed that kidney transplant senescence may contribute to graft loss [42]. Tubular frailty, a characteristic of aged kidneys, renders them more susceptible to ischemia, reperfusion injury, toxic damage, and subsequent inflammation, which causes a significant challenge for transplant recipients. However, in order to meet the growing demand, there has been a substantial increase in the proportion of donors aged ≥60 years, which has risen from 21% in 2000–2001 to 42% in 2016–2017 within the Eurotransplant senior program [40]. Aubert et al. reported an elevated incidence of graft loss in recipients who received organs from extended criteria donors, classified as individuals aged over 60 without comorbidities or over 50 with at least two conditions such as hypertension, death due to cerebrovascular accident, or serum creatinine levels exceeding 1.5 mg/dL [43]. The population of kidney transplant recipients represents a specific subgroup within the cohort of patients diagnosed with CKD who continue to face an elevated risk of progression to dialysis and mortality. Renal graft senescence is a major contributing factor that drives kidney transplant recipients towards ESRD.

## 5. The Common Mechanisms in the Regulation of Kidney Aging and CKD Progression

Increasing evidence has indicated that kidneys in aging and CKD share many common stressors, including senescence, autophagy, epigenetic and mitochondrial dysfunction, etc., suggesting that they may be regulated by similar underlying mechanisms. We will provide a comprehensive discussion about the common mechanisms that contribute to kidney aging and CKD.

### 5.1. The Roles of Senescence and Its Regulation in Kidney Aging and CKD

**Figure 1:**
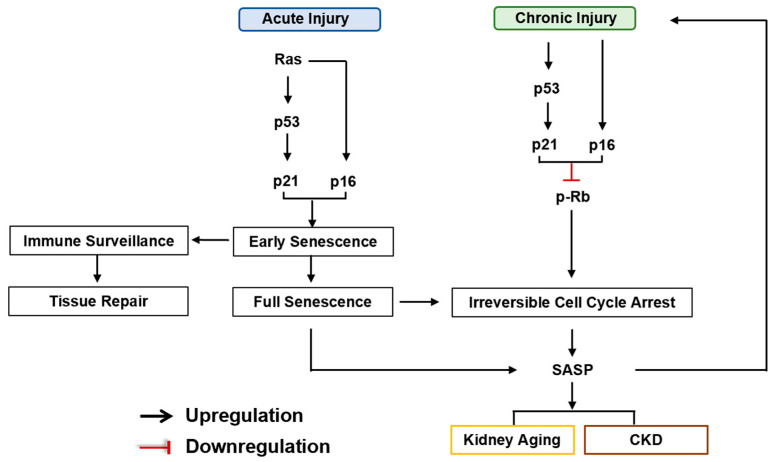
Multi-step senescence model in kidney aging and CKD. Acute injuries, such as wound, oxidative stress, and acute ischemia, can induce early cellular senescence via the activation of the p16^Ink4a^ and/or p53-p21 pathways. Early senescence causes the cell cycle to be temporarily blocked, which helps cells to avoid uncontrolled mitosis and provides more time for DNA repair. Meanwhile, activated immune cells can eliminate early senescent cells, which has been known as “immune surveillance”, and subsequently promote tissue repair. Prolonged exposure to stress-induced damage leads to a transition from early senescence to full senescence, resulting in the production of a senescence-associated secretory phenotype (SASP). Chronic injuries, which are induced by multiple and persistent stresses, lead to senescence and an irreversible cell-cycle arrest. The increasing numbers of senescent cells accumulate and induce more severe senescence by secretion of SASP, which eventually leads to kidney aging and CKD.

Cellular senescence is a process that is caused by a variety of stresses, leading to an irreversible and permanent cell cycle arrest coupled with the alteration in the transcriptome and secretome [44]. The process of cellular senescence can be categorized into two types: Acute senescence and chronic senescence [44]. Acute senescence plays a beneficial role by serving as a programmed mechanism that regulates embryogenesis, restricts tumorigenesis, facilitates wound healing, and enhances tissue repair [3]. Subsequently, the infiltrated macrophages can efficiently eliminate acute senescent cells in a timely manner. However, when the immune system fails to adequately clear the accumulating number of newly produced senescent cells, acute senescence inevitably disrupts tissue homeostasis [45]. Chronic senescence significantly blocks cellular renewal and proliferation, while it also triggers inflammation and fibrotic responses through the senescence-associated secretory phenotype (SASP). Chronic senescence can also evolve from acute senescence if immune clearance is impaired with age [46]. Senescence is considered to be a dynamic, multi-step process, with the senescent phenotype being influenced by the duration and intensity of injury, the transition from acute to chronic senescence, and irreversible cell cycle arrest Figure 1.

Senescent cells are increased and play a critical role in the aging process of the kidney [47]. The phenotypes of cellular senescence, including the activation of senescence-related pathways (p16^Ink4a^ and/or p21^Cip1^) and increased secretion of SASP, have been found in the aged kidneys. It has been reported that cellular senescence exhibits a progressive accumulation in the cortical tubules with advancing age, as seen by an increase in the expression of p16 and p21 in kidney tissues across different ages ranging from 21–80 years old [48]. Almost all human biopsy specimens exhibited positive p16^Ink4a^ staining in the nuclei of distal tubules and collecting ducts [6]. Because senescent tubular epithelial cells are present in 80% of diseased kidneys compared to only 21% in normal kidneys, they have been identified as a distinguishing factor between diseased and control kidneys [49]. Additionally, a comparable age-related augmentation of senescent cells is also detected in the glomeruli, tubulointerstitium, and arteries within the human kidney, as examined by p16 and SA-β-Gal staining [50]. The p16^INK-ATTAC^ mice, in which apoptosis of highly p16^INK-ATTAC^-expression cells can be induced upon administration of the drug AP20187 (AP), have been utilized to investigate the presence of p16 positive cells in kidneys during aging and renal fibrosis [51]. The ablation of senescent cells in aging p16^INK-ATTAC^ mice significantly ameliorated glomerulosclerosis, indicating the contribution of p16-positive cells to this prevalent hallmark of aging kidney [52]. Studies have also demonstrated an upregulation of p21 and SASP factors in aged mammalian kidneys, which contribute to the development of kidney injuries. Eliminating senescent cells or reducing the generation of SASP could ameliorate kidney inflammation and fibrosis in aging kidney [53].

Senescent cells are also increased and contribute to the development of CKD [54]. Acute injuries, such as wounds, oxidative stress, and acute ischemia, can induce early cellular senescence via the activation of the p16^Ink4a^ and/or p21^Cip1^ pathways. Early senescence causes the cell cycle to be temporarily blocked, which helps cells avoid uncontrolled mitosis and provides more time for DNA repair. Persistent injury leads to irreversible cell cycle arrest, ultimately promoting the transition from AKI to CKD. The accumulation of senescent cells was confirmed in the kidneys of fibrotic animal models (unilateral ureteral occlusion and ischemia-reperfusion-induced kidney injuries). Additionally, the accumulation of senescent cells and activation of the p16^Ink4a^ and/or p21^Cip1^ pathways have also been reported in patients with CKD.A retrospective clinical study suggests a correlation between cellular senescence and the kidney tubular epithelia of IgAN patients [55]. P16^Ink4a^-positive cells can be observed in the glomeruli and tubulointerstitium of renal biopsies from patients with minimal change disease (MCD), membranous nephropathy (MN), and FSGS [56]. An increase in p16^Ink4a^ and SA-β-gal in renal epithelial cells in biopsy specimens from patients with diabetic nephropathy (DN) suggests that senescence may also contribute to the progression of DN, one of the secondary glomerulonephropathies [57].

### 5.2. The Roles of Autophagy in Kidney Aging and CKD

Autophagy is a highly conserved lysosomal pathway responsible for the degradation of cytoplasmic components [58]. Depending on the organelles to be degraded and the route of cargo delivery to lysosomes, autophagy can be categorized into macroautophagy, microautophagy, or chaperone-mediated autophagy [59]. Macroautophagy involves the engulfment of large cytoplasmic materials by autophagosomes, which subsequently merge with lysosomes for degradation. Microautophagy involves directly trapping small cytosolic components into an inward invagination of the lysosomal membrane, without forming an autophagosome. Macroautophagy (hereafter termed autophagy) plays a crucial role in various fundamental biological processes [60].

Basal autophagy in renal cells plays a crucial role in maintaining kidney homeostasis, structure, and function [60]. Over the past decade, numerous studies have demonstrated the ability of autophagy to exert a protective effect against kidney aging [61]. The expression of autophagy proteins, such as light chain 3-I (LC3-I) and autophagy-related 7 (ATG7), is significantly decreased in the kidneys of 24-month-old rats compared to that in kidneys from 3-month-old animals, especially in proximal tubular cells and podocytes [62]. The specific deficiency of ATG5 in renal proximal tubules, which is a key regulatory gene in the autophagy process, enhances tubular apoptosis and tubulointerstitial fibrosis, ultimately leading to premature aging [63]. Although some studies suggest that basal autophagy in the proximal tubules of young mice is low, it is unable to increase in aging mice, which is attributed to age-related tissue hypoxia and the increase in cellular waste products [64]. Disruption of autophagy in proximal tubules in the kidneys of 24-month-old mice resulted in significant impairment of kidney function, along with tubular atrophy and tubulointerstitial fibrosis, suggesting that autophagy impairment accelerated the aging process and tubular damage [64].The impairment of autophagy in podocytes is also considered to promote aging. Podocytes, identified as the terminally differentiated postmitotic cells, have very high basal autophagy activity in order to maintain their capacity for eliminating protein aggregates and organelles [65]. The aging process is associated with a decline in autophagy within podocytes, which subsequently accelerates the generation of SASP, as evidenced by a targeted deletion of ATG5 in podocytes resulting in age-related alterations, including mitochondrial damage, endoplasmic reticulum stress, and accumulation of oxidized and ubiquitylated protein aggregates as well as lipofuscin in *Atg5*^Δ*podocyte*^ mice compared to control littermates [66].

The role of autophagy in CKD has been extensively investigated in experimental models, such as in unilateral ureteric obstruction (UUO) animals, or patients. It has been reported that autophagy is persistently activated in obstructed kidney tubules in UUO mice [67]. Inhibition of autophagy by chloroquine or selective deletion of ATG7 in proximal tubules reduced kidney fibrosis, respectively, possibly through attenuation of tubular apoptosis, macrophage infiltration, and fibroblast growth factor production. However, autophagy in renal distal tubular epithelial cells (TECs) also plays a protective role in the development of renal tubulointerstitial fibrosis through a recovery of the expression of LC-II and ATG7 and an inhibition of the protein kinase B (PKB, or AKT)-mammalian target of the rapamycin (mTOR) pathway after UUO injuries [68,69]. However, the role of autophagy in renal fibrosis is controversial, but most studies support its activation after acute injury and decline during chronic processes.

In specimens of human kidneys, autophagy was impaired in patients with diabetic kidney disease (DKD), FSGS, and polycystic kidney disease (PKD) [70]. The impaired autophagy observed in diabetes may result in a diminished ability to remove damaged lysosomes, leading to the accumulation of advanced glycation end products (AGEs) to further disrupt lysosomal function and ultimately contributing to the progression of DKD [71]. Patients with FSGS also exhibited a significantly reduced proportion of autophagosome-positive podocytes and lower levels of kidney Beclin 1 compared to patients with MCD [72]. Autophagic flux was found to be impaired in renal epithelial cells of PKD patients, indicating that this impairment may contribute to the progression of PKD [73].

**Figure 2:**
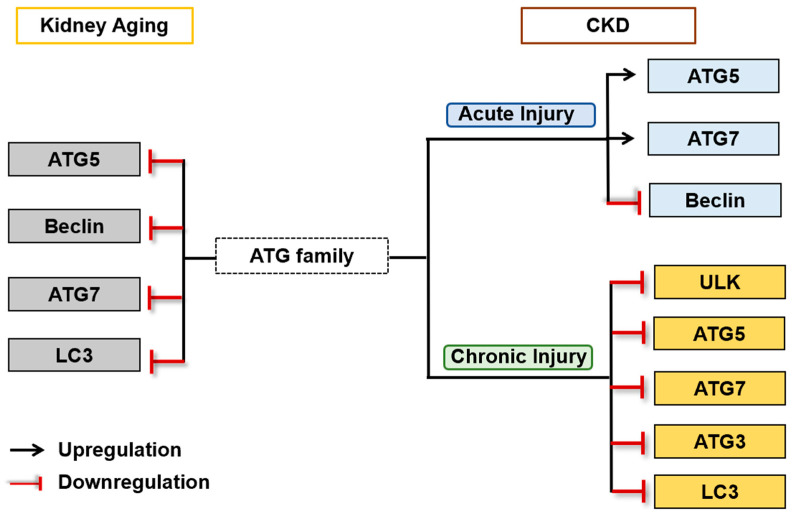
Autophagy-related ATG proteins involved into kidney aging and CKD. Numerous ATGs have been implicated in the regulations of kidney aging and CKD, which are grouped to indicate their contributions to kidney aging (gray), acute injury (blue) or chronic injury (yellow).

Collectively, studies suggest that autophagy plays a dual role in aging and CKD. Autophagy is suppressed in aging kidneys, which accelerates the progression of aging and age-related kidney diseases. However, in certain circumstances, constant activation of autophagy may lead to tubular atrophy and consequently contribute to the development of kidney fibrosis, whereas autophagy-mediated degradation of excessive collagen can potentially prevent fibrosis. More importantly, autophagy may interact with other cellular processes to exert an impact on the pathogenesis of aging and CKD, and further investigation is required to elucidate the underlying mechanisms. The ATGs currently reported to be involved in kidney aging and CKD are summarized in Figure 2.

### 5.3. The Roles of Mitochondrial Dysfunction in Aging and CKD

Mitochondria are intracellular organelles that possess an inner and outer membrane, with an intermembrane space separating them [74]. The outer membrane (OM) of mitochondria establishes connections between mitochondria and other cellular organelles, such as the endoplasmic reticulum (ER), lysosome, and plasma membrane. The inner mitochondrial membrane (IMM) exhibits a complex folded structure that significantly increases its surface area, which is divided into two compartments: The intermembrane space and the matrix. IMM forms the inner boundary membrane, which closely approximates the OM [75]. Within this intricate arrangement lies the cristae structure, housing respiratory chain components. The tightly packed ridges of cristae create enclosed regions essential for oxidative phosphorylation to generate ATP—an energy source—and house transport proteins regulating metabolite movement in and out of the matrix, where enzymes participate in tricarboxylic acid cycle (TCA) reactions and fatty acid cycles, along with DNA, RNA ribosomes, and calcium granules [74,75].

Kidneys are one of the most energy-demanding organs, with a high abundance of mitochondria present in renal tubule cells. Different nephron segments exhibit variations in mitochondrial densities and distributions due to diverse energy requirements [74]. Renal proximal tubular cells necessitate substantial energy for reabsorption and secretion against chemical gradients, while podocytes, endothelial cells, and mesangial cells possess greater flexibility in their glycolytic capacity for energy generation [74]. The maintenance of normal mitochondrial structure and function is crucial for cellular homeostasis during aging and CKD. Various endogenous and exogenous injuries may lead to mitochondrial dysfunction, mitochondrial-mediated inflammation, and decreased mitophagy, ultimately promoting the progression of aging and CKD [76].

#### 5.3.1. Mitochondrial Function in Kidney Aging and CKD

The primary biological function of mitochondria is to generate a substantial amount of adenosine triphosphate (ATP), thereby supplying the energy source for basal cell functions in the kidneys [77]. Increasing studies have revealed the role of mitochondrial dysfunction in the pathogenesis of both kidney aging and CKD. The aging process is accompanied by a reduced capacity of mitochondria to generate adenosine triphosphate (ATP) via the oxidative phosphorylation pathway, leading to insufficient cellular energy supply for adequate metabolic activities [78]. Ras-senescent cells were dysfunctional, with compromised ATP generation due to the continuous oncogenic stress. Consequently, mitochondrial function gradually deteriorates, leading to an impairment of cell activity and an increase in cellular senescence [79]. The renal cortex of aged rats exhibited impaired mitochondrial function compared to the control group [80]. It has been reported that upregulation of Klotho restored mitochondrial functional homeostasis and enhanced the expression of subunits in the mitochondrial respiratory chain complex, as evidenced by the detection of translocase of outer mitochondrial membrane complex subunit 20 (TOMM20) and oxidative phosphorylation (OXPHOS) complex III subunit cytochrome b (Cytb), thereby improving kidney aging and fibrosis [81]. The expression of cannabinoid receptor 2 (CB2), responsible for activating the endocannabinoid system, is upregulated in the kidney during aging and coincides with a decline in mitochondrial mass. Deletion of the CB2 gene in aging mice significantly inhibits β-catenin signaling activation, leading to the restoration of adenosine triphosphate production, thereby ameliorating the kidney aging phenotype [82]. These findings suggest that restoring the capacity of mitochondrial ATP generation function can effectively inhibit the progression of kidney aging.

The inhibition of fatty acid oxidation results in a metabolic shift towards glucose utilization and decreased ATP production in CKD [83,84]. The expression of rate-limiting enzymes involved in fatty acid oxidation and glycolysis was upregulated in the proximal tubules of a mouse model with subtotal nephrectomy (STN), while mitochondrial respiration was reduced [85]. These findings suggest that proximal tubular mitochondrial respiration is suppressed and ATP synthesis is altered in the CKD model induced by subtotal nephrectomy. Metabolic reprogramming is another factor that impacts mitochondrial ATP function in patients with CKD. Lipid deposition is associated with the downregulation of several key genes involved in fatty acid oxidation, thereby linking the inhibition of fatty acid oxidation to lipid accumulation [84]. A genome-wide transcriptomic analysis of a large cohort of normal and fibrotic human kidney samples revealed decreased expression levels of carnitine palmitoyltransferase-1/2 (CPT-1/2) and other mitochondrial genes in the fibrotic samples [86].

Currently, many studies target protecting mitochondria to recover ATP synthesis through oxidative phosphorylation, thereby counteracting the impact of certain aging factors and ultimately ameliorating the aging process [87]. It is also important to recover ATP synthesis in CKD patients, particularly during the AKI-CKD transition and in CKD cases related to metabolism [74].

#### 5.3.2. Mitochondria-Mediated ROS and Inflammation in Kidney Aging and CKD

##### Mitochondria-Mediated ROS in Kidney Aging and CKD

Reactive oxygen species (ROS) are generated by various cellular compartments, including the cytoplasm, cell membrane, endoplasmic reticulum, mitochondria, and peroxisome. Among these compartments, mitochondrial reactive oxygen species (mtROS) play a predominant role in the production of ROS, accounting for approximately 90% of cellular ROS generation [88].

Many studies found that ROS levels were increased in the elderly mammalian kidneys compared with those in young individuals [89]. The depletion of mitochondrial superoxide dismutase and catalase results in an elevation of oxidative stress in mice, promoting kidney aging [90]. Additionally, the increased production of mtROS disrupts the cell membrane lipids and reduces levels of fatty acids, ultimately leading to an acceleration in cellular senescence [91]. ROS is generated by nicotinamide adenine dinucleotide phosphate (NADPH) oxidases [92]. Elevated NADPH oxidase activity may serve as an early indicator for predicting future oxidative stress in renal tissue during senescence-accelerated mouse prone-8 (SAM-P8) mice, which undergo age-related changes [93]. The depletion of isocitrate dehydrogenase 2 (IDH2) (mitochondrial NADP^+^-dependent isocitrate dehydrogenase) has been shown to expedite kidney aging in idh2^−/−^ mice [94]. Activation of the nuclear factor erythroid 2-related factor 2 (Nrf2) and Sirt1 signaling pathways in the kidneys of aging mice has been demonstrated to alleviate renal oxidative stress and mitochondrial dysfunction [95].

Mitochondrial oxidative stress is also increased in CKD kidneys compared to healthy kidneys. In a cohort study, Oberg et al. found that patients with CKD (stage 3–5) exhibited elevated levels of oxidative stress compared to the healthy subject cohort [96]. Additionally, an increase in oxidative stress was noted in diabetic and hypercholesterolemic patients. The increase in oxidative stress in CKD is associated with mitochondrial respiratory dysfunction, which can be both the consequence and the cause of heightened oxidative stress, as determined by employing a high-throughput genomic approach based on comprehensive transcriptomic analysis combined with classical molecular methodologies [97]. Experimental evidence using a redox-sensitive green fluorescent protein biosensor (roGFP) confirmed a substantial rise in mitochondrial reactive oxygen species within CKD kidneys [74]. Treatment with mitochondria-targeted antioxidants such as mitoTEMPO demonstrated potential for alleviating podocyte injury and loss associated with diabetic nephropathy [98].

##### Mitochondria Dysfunction-Mediated Inflammation in Kidney Aging and CKD

Mitochondria dysfunction can trigger cellular inflammatory responses through several pathways. Firstly, inflammation is the predominant consequence of oxidative stress; these two processes are intricately intertwined in a detrimental cycle [99]. Oxidative stress triggers the activation of transcription factors such as NF-ҡB, which promotes the expression of proinflammatory cytokines and chemokines and then the recruitment of immune cells [100]. The recruited leukocytes and macrophages release proinflammatory oxidized lipids and advanced protein oxidation products, ultimately accelerating oxidative stress. Secondly, the leakage of large amounts of mitochondrial contents can directly activate inflammatory responses. Mitochondrial DNA (mtDNA) could activate the NLRP3 inflammasome, which in turn facilitates IL-1β and IL-18-mediated inflammation [101]. Finally, mtDNA in the cytoplasm can be recognized by the cytoplasmic sensor cyclic guanosine monophosphate–adenosine monophosphate (GMP–AMP) synthase (cGAS) and activates the transcription factor interferon regulatory factor 3 (IRF3) via stimulator of interferon genes (STING) and TANK binding kinase 1 (TKB1), promoting inflammation [102]. Collectively, mitochondria play an essential role in cellular and inflammatory responses.

Kidney mtDNA was the most susceptible to the accumulation of age-related damage, whereas liver, testis, and lung were the least susceptible organs [103]. MtDNA mutator mice, carrying a defective proofreading function in their nuclear-encoded mtDNA polymerase, show accelerated accumulation of random mutations in mtDNA and exhibit marked features of premature aging. It was reported that PINK1 contributed to kidney aging via the cGAS-STING pathway, which is known to result in an inflammatory phenotype and disruption in mitochondrial metabolism [104]. The process of kidney aging is associated with a significant increase in inflammasomes, as evidenced by the upregulation of pyrin domain-containing protein 3 (NLRP3), leucine-rich repeat-containing receptor (NLR) required for cell death 4 (NLRC4), and pro-caspase-1 levels in the kidneys of 24-month-old rats [105]. More importantly, caloric restriction is recognized as an important way to prevent the progression of age-related kidney disease by inhibiting mitochondrial-induced inflammation [106].

Several studies have demonstrated the important role of mitochondria in the regulation of inflammation during CKD [74]. The cGAS-STING pathway is activated in response to the presence of mtDNA within the cytosol of cells in murine models of renal fibrosis. Inhibition of STING by using C176, or genetic deletion of STING, attenuated kidney injury in chronic kidney disease models. NLRP3 has recently been reported as a central pathogenic mechanism of CKD. NLRP3 recruits and activates caspase-1, which cleaves the cytokines pro-IL-1β and pro-IL-18 into their mature and biologically active forms. NLRP3 inflammasome and its downstream cytokines increased in UUO mice, db/db mice, and IRI-injured mice kidneys [107]. Additionally, it also revealed an upregulation in the expression of the NLRP3 inflammasome within the renal tubules of patients with diabetic and IgA-associated tubulointerstitial injuries [108].

Persistent oxidative stress and low-grade inflammation are hallmarks of the uremic phenotype, contributing to aging kidney and CKD. Growing evidence demonstrates that mitochondria function as a central regulator, checkpoint, and arbitrator in triggering ROS and inflammation in response to various stimuli. Notably, mitochondrial ROS and mito-inflammation reciprocally influence each other, synergistically promoting disease progression. Targeting mitochondria holds potential for ameliorating kidney aging and CKD [109].

#### 5.3.3. Mitophagy Mediated Mitochondrial Elimination in Kidney Aging and CKD

Mitophagy-mediated elimination of mitochondria plays an important role in many processes [110]. Mitophagy is defined as the selective degradation of excessive or damaged mitochondria through the lysosome pathway [111]. Mitophagy is predominantly mediated by LC3-associated autophagy receptors via both Ub-dependent and Ub-independent pathways [112]. Ub-dependent mitophagy involves the mitochondrial serine/threonine protein kinase PINK1 and the E3 Ub-protein ligase parkin (PINK1–parkin) pathway [113]. Ub-independent mitophagy is mediated by various outer mitochondrial membrane (OMM) receptors that do not rely on interaction with ubiquitin for cargo recognition.

The continuous accumulation of dysfunctional mitochondria is a crucial hallmark of aging, and the process of abnormal mitophagy plays an essential role in eliminating damaged mitochondria during the aging process [114]. It has been reported that mitophagy is significantly reduced during physiological aging [115]. The expression of legumain, a conserved lysosomal peptidase, has been reported to be significantly downregulated in the kidney with advanced age. Deficiency of legumain induces tubular cell senescence due to impaired clearance of damaged mitochondria in renal tubulo-specific legumain-knockout mice or legumain-knockdown HK-2 cells [116]. Mechanistically, the downregulation of legumain could potentially enhance lysosomal function, thereby facilitating the timely clearance of damaged mitochondria. Evidence indicates abnormal lysosome function and impaired mitophagy in tubule cells of aged legumain-knockdown mice [116]. The expression of Gli-like transcription factor 1 (GLIS1), which is associated with mitophagy, is decreased in both natural and accelerated aging kidney models [117]. In addition, the recovery of mitophagy and the delay in renal aging were observed following calorie restriction in rat models [118]. These findings reveal the importance of maintaining mitophagy in kidney aging, and maintaining mitophagy activity is conducive to the timely removal of damaged mitochondria.

The role of mitophagy has been specifically implicated in several kidney diseases. The expression of PINK1 (Ub-dependent mitophagy pathway), mitofusin 2 (MFN2), and Parkin was significantly decreased in the experimental models of kidney fibrosis as well as in patients with CKD [119]. Choi et al. have delineated the cytoprotective role of PINK1/Parkin-dependent mitophagy against kidney fibrosis [120]. Other studies found that mitophagy was increased in kidneys following UUO or contrast-induced acute kidney injury [121]. Mitophagy was abolished when silencing PINK1 or Parkin, indicating a dominant role of the PINK1–Parkin pathway in mitophagy. Renal injuries under UUO or contrast exposure were more severe in PINK1- or PARK2-deficient mice than in wild-type groups [121]. The renoprotection of mitochondrial autophagy has been widely explored in the context of DN and the transition from AKI to CKD [74].

**Figure 3:**
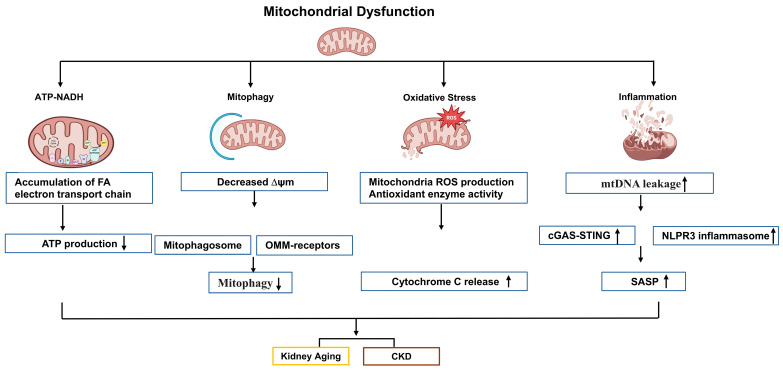
Schematic illustration of pathophysiological processes of mitochondrial dysfunction, including disrupted of ATP production, mitophagy, oxidative stress, and inflammation, in the process of kidney aging and CKD. Here, we summarize the common pathways underlying mitochondrial dysfunction in aging kidney and CKD. The main biological function of mitochondria is to generate a significant amount of ATP. Stressors disrupt the respiratory chain, leading to an imbalanced NAD+/NADH ratio, which impairs ATP production and mitochondrial membrane potential (MMP). Additionally, dysregulated lipid accumulation decreases ATP production through oxidative phosphorylation. The reduction in mitophagy in senescence disrupts cellular homeostasis by impairing the degradation of damaged mitochondria. Mitophagy is predominantly mediated by decreased MMP via Ub-dependent and Ub-independent pathways. The process of Ub-dependent mitophagy involves the formation of the mitophagosome, whereas Ub-independent mitophagy is facilitated by various OMM receptors. Oxidative stress and inflammation are considered common features of aging kidney and CKD. The activation of mitochondrial ROS and the inactivation of antioxidant enzymes can cause cytochrome c to be released into the cytosol, ultimately triggering cell inflammation and apoptosis. Mitochondria-induced inflammatory responses activate NLRP3 inflammasome signaling as well as the cGAS-STING pathway following mtDNA leakage. CKD: chronic kidney disease; NAD: nicotinamide adenine dinucleotide; NADH: nicotinamide adenine dinucleotide; ATP: adenosine triphosphate; ΔΨm: mitochondrial membrane potential; OMM: outer mitochondrial membrane; ROS: reactive oxygen species; mtDNA: mitochondrial DNA; cGAS; cyclic guanosine monophosphate–adenosine monophosphate (GMP–AMP) synthase; STING: stimulator of interferon genes; NLRP3: pyrin domain (PYD)-containing 3; SASP: senescence-associated secretory phenotype; ↑: upregulation; ↓: downregulation.

Taken together, mitochondrial dysfunction contributes to kidney aging and CKD through a common pathway involving reduced ATP production, impaired mitophagy, and the induction of oxidative stress and inflammation, as summarized in Figure 3. However, it remains unclear whether mitophagy is a consequence of the mitochondrial fission/fusion mechanism or an independent event. Further studies are needed to elucidate the involvement of the mitochondrial fission/fusion machinery in regulating mitophagy during aging and CKD.

### 5.4. The Roles of Epigenetic Regulation in Kidney Aging and CKD

Aging and CKD are accompanied by alterations in epigenetic regulation, including DNA methylation, histone modification, chromatin remodeling, regulation of non-coding RNA (ncRNA), and RNA modification. We summarize the epigenetic mechanisms that have been reported to regulate the process of aging and CKD.

#### 5.4.1. DNA Methylation in Kidney Aging and CKD

DNA methylation takes place at the cytosines within CpG dinucleotides, resulting in the formation of 5-methylcytosine (5-mC). It is estimated that approximately 60–90% of CpG sites undergo methylation in the mammalian genome [122]. During the process of aging, there is a general trend towards hypomethylation of the genome. The methylation of DNA is mediated by DNA methyltransferases (DNMTs), while the methyl group on DNA is removed by ten-eleven translocation (TET) [123]. Age estimators such as Horvath’s clock, Hannum’s clock, PhenoAge, GrimAge, and single-cell age clock (scAge) are based on alterations in DNA methylation patterns within the genome [124].

Several genome-wide association studies (GWASs) and epigenome-wide association studies (EWASs) identified significant changes in DNA methylation with aging and age-related kidney diseases [125,126]. A comprehensive analysis of DNA methylation changes across the entire genome, covering over 800,000 CpG sites, in 95 renal biopsies obtained from donors aged between 16 and 73 years prior to kidney transplantation found a strong link between the aged kidney and methylated regions, suggesting that changes in DNA methylation play a role in kidney injury damage during the aging process [125]. It has also been shown that an accumulation of uremic toxins leads to DNMT-mediated repression of the expression of Klotho, an anti-aging single-pass membrane protein [127]. In addition, the upregulation of DNMT1/3a/3b is associated with a hypermethylation in the promoters of the Klotho gene both in natural aging kidneys and D-galactose-induced aging kidneys, whereas treatment with the DNMT inhibitor, SGI-1072, effectively hypomethylates the promoter of Klotho, restoring Klotho expression and improving kidney aging [128].

DNA methylation is also involved in the occurrence and progression of CKD. Profibrotic factors, such as TGF-β, can induce the upregulation of DNMT1/3a/3b, suppressing the expression of Klotho in CKD mice [129]. In contrast, the restoration of TET activity can reverse the methylation of the Klotho promoter, thereby alleviating the progression of CKD [130].

#### 5.4.2. Histone Modification in Kidney Aging and CKD

The modification of histones, including methylation, acetylation, phosphorylation, ubiquitination, and ADP ribosylation, can either activate or suppress gene expression and regulate kidney aging and CKD. Among these modifications, lysine residue methylation and acetylation have been extensively studied in the aging process and CKD [131].

##### Histone Acetylation in Kidney Aging and CKD

Histone acetylation is regulated by histone acetyltransferases (HATs) and histone deacetylases (HDACs) [132].

HATs and HDACs have been implicated in the regulation of kidney aging and CKD [133]. In particular, studies have been focused on the roles of HDACs in kidney aging [133]. The aberrant activation of HDAC3 contributes to kidney aging by impairing the process of autophagy [134]. In addition, it has been demonstrated that HDAC3 inhibits the transcription of Klotho [135]. The class III nicotinamide adenine dinucleotide (NAD^+^)-dependent HDACs, sirtuins (Sirts), are widely recognized as critical regulators of aging. SIRT1 is significantly decreased during kidney aging, which correlates with alterations in the expression profiles of multiple target molecules. Podocyte-specific silencing of SIRT1 could promote age-related glomerulosclerosis [136]. Endothelial Sirt1 deficiency contributes to nephrosclerosis, which is primarily linked to renal vascular aging [137]. SIRT3 has been reported to act as an essential regulator of cell senescence. Angiotensin II (Ang II) type 1 receptor (AT_1_R) deletion upregulated SIRT3 expression in the kidneys of aged mice. However, other studies reported that SIRT1, not SIRT3, was significantly reduced in the kidneys of aged mice with AT_1_R-associated protein (ATRAP) deletion [138]. Nevertheless, Sirt3 deficiency is known to cause severe renal fibrosis in aging kidneys through an increase in transforming growth factor-β1 (TGF-β1) and hyperacetylation of glycogen synthase kinase-3β (GSK-3β) [139]. In addition, activation of Sirt6 could attenuate age-associated kidney injury by inhibiting the proinflammatory NF-ҡB signaling pathway [138].

Studies have also demonstrated that histone acetylation participates in renal fibrosis of CKD [140]. P300/CBP-associated factor (PCAF) has histone acetyl transferase activity and regulates the molecular machinery of renal fibrosis and inflammation [140]. The expression of PCAF is increased in UUO-induced kidney injury and is coincident with an activation of NF-κB signaling [141]. In addition, HDACs are also involved in the regulation of renal fibrosis. HDAC2 has been shown to induce apoptosis of renal tubular epithelial cells and mediate renal fibrosis in diabetic mice and IR-treated mice [142]. HDAC3 was elevated in fibrotic kidneys after UUO and aristolochic acid nephropathy (AAN) injury [143]. Treatment with trichostatin A (TSA), a pan inhibitor of class I and II HDACs, significantly inhibited epithelial-mesenchymal transdifferentiating (EMT) and attenuated renal fibrosis by downregulating TGF-β_1_ expression and inhibiting the JNK/Notch-2 signaling pathways [140]. HADC4, a class IIa HDAC, has also been upregulated in the progression of renal fibrosis following UUO injury [144]. HDAC6 is the most studied isoform of class IIb HDACs in kidney diseases. Unlike other HDAC isoforms, whose deletion in mice leads either to death in utero or severe developmental defects, mice with HDAC6 deletion develop normally without major organ dysfunction [140]. Inhibition of HDAC6 with specific inhibitors offers a promising therapeutic approach for inhibiting renal fibrosis. HDAC 11 is the sole isoform of class IV HDACs. A recent study revealed that the expression of HDAC11 was also increased in the kidney of murine models of CKD induced by UUO, Ang II, or a high-fat diet [145]. In contrast, Sirts has been proven to exert a renoprotective role in CKD. Blockage of Sirt6 deacetylase activity aggravates renal fibrosis induced by UUO and renal ischemia-reperfusion (IR) [146]. Mechanistically, Sirt6 interacts with β-catenin and subsequently deacetylates H3 lysine 27 (H3K56), thereby exerting a negative regulation on the β-catenin pathway [146].

##### Histone Methylation in Kidney Aging and CKD

The methylation of histones represents another significant histone modification. Histone methylation is regulated by lysine methyltransferases (KMTs) and lysine demethylases (KDMs) [147].

Histone methylation modification is involved in age-related gene regulation. H3K27me3 levels were increased in the aged mouse kidneys. Elevation of H3K27me3 levels was likely due to the downregulation of H3K27-specific demethylase JMJD3 and H3K27-specific methyltransferase enhancer of zeste homolog 2 (EZH2) [148]. H3K27me3 was associated with the Klotho promoters. Inhibition of EZH2 resulted in a reduction in H3K27me3 levels, leading to enhanced Klotho expression both in vivo and in vitro [148]. SET domain-containing 2 (SETD2) is responsible for the trimethylation of histone H3 lysine36 (H3K36me3). The SETD2 knockdown renal primary tubular epithelial cells (PTECs) maintained a low level of CDKN2A and no β-galactosidase staining, confirming the protective role of SETD2 against senescence [149].

The expression of EZH2 was significantly increased primarily in the proximal tubules and myofibroblasts of UUO kidneys [150]. Inhibition of EZH2 effectively suppressed H3K27 methylation and prevented extracellular matrix protein deposition in UUO mouse kidneys [150]. However, advanced glycation end products (AGEs) could decrease EZH2 expression in podocytes, leading to a reduction in H3K27me3 [151]. This suppression of EZH2 mimicked the AGEs effects and caused an upregulation of the expression of pathological factors that contribute to podocyte injury in DKD [151]. The expression of LSD1 is also upregulated in mouse kidneys with UUO injury and in NRK-52E cells treated with TGF-β1. Inhibition of LSD1 with its specific inhibitor, ORY1001, attenuated renal fibrosis, accompanied by an increase in H3K4me1/2 [152]. SET and MYND domain protein 2 (SMYD2) is a lysine methyltransferase that mediates H3K36me3, which was highly expressed in interstitial fibroblasts and renal tubular epithelial cells of mouse kidneys with UUO injury. Inhibition of SMYD2 with AZ505 protected against renal fibrosis and inhibited the activation of renal interstitial fibroblasts [153].

##### Non-Coding RNAs in Kidney Aging and CKD

Other epigenetic mechanisms include non-coding RNA (ncRNA) transcripts in epigenetic gene regulation. Increasing studies have uncovered the roles of microRNAs (miRNAs) (single-stranded transcripts consisting of approximately 22 nucleotides) and long ncRNAs (lncRNAs) with a length exceeding 200 nucleotides in kidney aging and CKD [154].

It has been reported that the levels of miR-21 and miR-200c are increased in the renal cortex with aging [155]. The upregulation of miR-21 and miR-200c was associated with a decrease in the expression of their respective targets, Sprouty-1 and zinc finger E-box binding homeobox 2 (ZEB2), which regulate the transcription of collagen I and III, leading to an elevation in the expression of collagen I and III during the aging process. Studies have also reported that lncRNAs are involved in kidney aging. LncRNA-ATB enhances inflammation, cell apoptosis, and senescence in TGF-β1-induced HK-2 cells through the TGF-β/Smad signaling pathway [156]. LncRNA SNHG21 binds to miR-25-3p and negatively regulates SIRT6 in Ang II-induced hypertensive mice [157]. LncRNA HOTAIR reduces the levels of the cell cycle inhibitor by inducing H3K27me3 methylation [158]. RNA-seq analysis reveals that lncRNA Gm43360 downregulates the expression of p53, p21, and SA-β-gal in naturally aged mouse kidneys [159].

The roles of miRNAs and lncRNAs in CKD have been extensively investigated over the past decades. Accumulating evidence suggests that TGF-β1 can modulate multiple miRNAs to promote renal fibrosis. Specifically, TGF-β1 upregulates miR-21, miR192, miR-377, miR-382, and miR-491-5p but downregulates the expression of miR-29 and miR-200 during renal fibrosis [160]. The role of miR192 in fibrosis remains controversial. miR192 is increased in mouse models with fibrosis and TGF-β1-treated murine cells [10], and knockout of miRNA192 significantly decreases renal fibrosis, possibly through an induction of zinc finger E-box binding homeobox 1/2 (ZEB1/2) [161]. However, other studies indicate that TGF-β1 reduces the expression of miRNA192 in human tubular epithelial cells (TECs), and deficiency of miRNA192 accelerates renal fibrosis in diabetic nephropathy [160]. In terms of lncRNAs, 21 novel Smad3-dependent lncRNAs were identified using high-throughput RNA sequencing in two mouse models: UUO-induced nephropathy and immunologically induced anti-glomerular basement membrane glomerulonephritis [162]. These newly discovered lncRNAs may contribute to the development of renal inflammation and fibrosis in injured kidneys, including DN, IgA nephropathy, anti-GBM nephropathy, ischemic renal disease, etc. [163].

In sum, our current understanding of DNA and histone modifications and non-coding RNAs in aging kidney and CKD is fragmentary and still in the infant stage. However, the constant identification of epigenetic regulators involved in kidney aging and CKD should provide potential therapeutic targets for improving kidney aging and CKD.

## 6. Therapeutic Strategies for Kidney Aging and CKD

Over the past few decades, a plethora of therapeutic approaches have been developed to delay the aging process and alleviate CKD progression, encompassing both pharmacological and non-pharmacological interventions. In particular, targeting senescence with senostatics and senolytics shows beneficial effects on aging and CKD. In addition, new strategies are constantly developed to delay aging and CKD. The therapeutic intervention for kidney aging is summarized in Table 1. Most of those agents targeting aging kidneys have been implemented in animal models of CKD.

### 6.1. Targeting Senescent Cells Delays Aging and Alleviates CKD

#### 6.1.1. Senostatics

Senostatics, also known as senomorphics, refer to drugs that possess the ability to interfere with the progression of cells entering senescence or modulate their activity by reducing the generation of SASP [164]. Rapamycin, a specific inhibitor of mTOR, has been shown to stimulate intracellular repair and extend life span via activating autophagy and improving mitochondrial complex I function [165]. Serval studies have demonstrated that inhibition of mTOR could reverse kidney aging in aged animals via enhancing autophagy and elevating the expression of Klotho [166]. Rapamycin also shows beneficial effects by reducing UUO-induced renal hypoxia, inflammation, and tubulointerstitial fibrosis in CKD animals [167]. Clinically, rapamycin is often prescribed as an immunosuppressive drug to prevent rejection after kidney transplantation [168]. However, the use of rapamycin as an anti-aging drug is limited due to its side effects, including insulin resistance, glomerular dysfunction, dyslipidemia, anemia, leucopenia, and thrombocytopenia [169].

Metformin has also been shown to extend lifespan through diverse pathways, including the activation of adenosine monophosphate-activated protein kinase (AMPK), the inhibition of the mechanistic target of rapamycin complex 1 (mTORC1), and the remodeling of complex I in the mitochondrial electron transport chain [102]. Metformin demonstrates efficacy in attenuating various hallmarks of aging, including improving nutrient sensing, enhancing autophagy, delaying stem cell aging, modulating mitochondrial function, regulating transcription, and reducing telomere attrition [170]. Administration of 1% metformin biweekly to the 2-year-old mice, which were fed with a standard chow diet, could improve some metabolic markers of health [171]. Metformin is currently being tested in a large clinical trial known as TAME (Targeting Aging by MEtformin) to evaluate its age-targeting effects [172].

In the past few decades, the renal protective effects of metformin have been reported in preclinical and clinical studies. Metformin effectively suppressed renal fibrosis and improved renal function in an adenine-induced nephrectomy and UUO mouse kidneys [173]. Neven et al. demonstrated that treatment with metformin decelerated the progression of advanced CKD and reduced the risks associated with vascular calcification [174]. In addition, treatment with metformin delays the progression of DKD by modulating metabolic dysfunctions, including insulin resistance, autophagy, oxidative stress, ER stress, and inflammation [174]. However, the renal effects of metformin are complex and dependent on the disease type. Thus, to establish the clinical efficacy of metformin, well-designed randomized controlled trials with larger sample sizes and longer durations are necessary.

The Janus kinase (JAK)/signal transducer and activator of transcription (STAT) pathway plays a crucial role in regulating SASP production; targeting JAK/STAT with inhibitors emerges as an appealing strategy for senomorphic intervention [175]. Treatment with JAK inhibitors decreased the expression of mRNAs of key SASP components, including IL-6, IL-8, and matrix metalloproteinase, in senescent cells; however, no impact was observed on non-senescent cells [176]. Additionally, administration of a selective JAK1/2 inhibitor resulted in a significant reduction in cytokine expression levels in aging mice compared to the control mice treated with vehicle. JAK/STAT signaling has also been found to be upregulated in all kinds of damaged kidneys, including lupus nephritis, DN, and IgAN [177]. Treatment with the inhibitor of JAK3 (CP690,550) could alleviate renal fibrosis in UUO mice [178]. However, the JAK/STAT inhibitor, tofacitinib, failed to mitigate inflammation induced by uric acid crystals and did not impede the progression of CKD [179]. The conflicting data from these studies suggest that JAK/STAT3 may have a dual role in kidney aging and CKD.

#### 6.1.2. Senolytics

Senolytics are drugs that specifically target senescent cells by inducing apoptosis of those cells but have no effect on non-senescent cells [180]. It has been reported that a combination of dasatinib and quercetin (D+Q) exhibits a broad spectrum of senolytic activity in eliminating senescent cells by transiently disabling pro-survival networks, including ephrin dependence receptor signaling, phosphoinositide-3-kinase–protein kinase B, and B-cell lymphoma-2 (BCL2) members [181]. Treatment with D+Q resulted in a significant 36% increase in median lifespan in aging mice [181]. In addition, treatment with D+Q was found to alleviate natural aging and CKD in mice induced by 5/6th nephrectomy [182]. In order to evaluate the efficacy of intermittent D+Q in targeting senescent cells in humans, oral administration of D+Q was conducted on DKD patients. The results showed that treatment with D+Q can alleviate insulin resistance, proteinuria, and renal podocyte dysfunction induced by a high-fat diet or genetic obesity in mice [183]. The beneficial effects of D+Q therapy on diabetes and CKD are currently being tested in an ongoing clinical trial (NCT02848131) [181].

The forkhead box subgroup 4 (FOXO4) peptide is a potent remedy that disrupts the interaction between FOXO4 and p53, leading to the selective exclusion of p53 from the nucleus and intrinsic apoptosis in senescent cells [47]. It has been demonstrated that FOXO4 peptide effectively restores renal function in naturally aging mice. AGEs accumulate in patients with diabetes and induce podocyte apoptosis by activating the FOXO4 transcription factor, suggesting that targeting FOXO4 may also hold promise for the treatment of DKD [184].

The compound ABT-263 (and related compounds ABT-737 and ABT-199), also known as navitoclax, can effectively inhibit the upregulation of BCL-2 family members in senescent cells, thereby overcoming their resistance to apoptosis [185]. Depletion of senescent cells with ABT-263 reduced irradiation-induced kidney injury in aging mice [186]. In addition, treatment with ABT-263 eliminated senescent mouse proximal tubular (BUMPT) cells with repeated low-dose cisplatin treatment and reversed the profibrotic phenotype of these cells [187].

The elimination of p16-positive senescent cells in both the p16-3MR (tri-modal reporter) and INK-ATTAC models also prevents age-related disorders. In particular, the elimination of senescent cells by suicide gene-mediated ablation of p16^Ink4a^-expressing senescent cells in INK-ATTAC mice delayed age-related deterioration of several organs, including kidney [51]. In addition, inhibition of kidney-type glutaminase (KGA)-dependent glutaminolysis in aging mice prolongs lifespan and demonstrates therapeutic benefits in age-associated tissue degeneration [188]. Similarly, increasing studies have shown a close association between P16^Ink4a^-positive senescent cells and CKD. Renal tubule-specific expression of P16^Ink4a^ is tightly correlated with blood glucose levels, while glomerular p16 expression is associated with proteinuria in patients with DKD. Westhoff et al. found an increase in kidney P16^Ink4a^ expression in patients with hypertensive nephropathy [189]. An association between the increase in p16^Ink4a^-positive senescent cells and the development of renal fibrosis was observed in IgAN patients who underwent kidney transplant [190]. These studies suggest that senolytics can be employed as a therapeutic strategy to effectively delay both the natural aging process of the kidneys and the progression of CKD and other kidney diseases.

### 6.2. The Development of New Strategies to Delay Kidney Aging and CKD Progression

Studies in aged mice have indicated that the cell cycle activity of hematopoietic stem cells (HSCs) decreases significantly, with old HSCs undergoing fewer cell divisions, regenerative capacity, and function than young HSCs [191]. Increasing evidence supports the presence of multipotent stem cells in the kidneys [192]. Fogo’s group reported that elderly mice that received young bone marrow presented less SA-β-gal staining of senescent cells than those mice that received old bone marrow [193]. Recently, engineered HSCs have been generated and demonstrated enhanced efficacy in treatment with anti-senescence. It is reported that human mesenchymal stromal/stem cells (MSC)-derived exosomes antagonize senescence in murine renal primary tubular epithelial cells (PTEC) [194]. In addition, an anti-KIM1 antibody coating MSC (KIM-MSC) was reported to enhance the ability of MCS to abrogate p16^Ink4a^ cells in murine renal artery stenosis [195]. Noh et al. found that uremic MSCs exhibited enhanced cellular senescence, reduced proliferation, impaired migration capacity, and compromised tube formation ability [196]. At present, MSC have shown evident therapeutic effects on AKI, CKD, DKD, and atherosclerotic renovascular disease (ARVD); however, their roles in the transplanted kidney remain controversial [197]. The efficacy of stem cell utilization in the treatment of CKD has been validated in clinical studies [198].

Chimeric antigen receptor T (CAR-T) cells, T-lymphocytes engineered to express a specific receptor to a targeting cell, possess the potential to act as a senolytic agent [180]. Amor et al. identified a novel urokinase-type plasminogen activator receptor (uPAR) as a cell-surface protein and found uPAR-specific CAR-T cells efficiently ablate senescent cells in lung adenocarcinoma and liver fibrotic animal models [199]. Not many studies have covered the repercussions of CAR T-cell therapy on the kidneys. Anwer et al. reported that 18% of patients developed AKI after receiving CAR-T cells; however, most cases suffered from hematologic malignancies. Thus, the application of CAR-T cells necessitates further investigation in kidney aging and CKD [200].

### 6.3. Lifestyle Modification

Lifestyle modification is the only practical and probable strategy to promote health and longevity in humans. Calorie restriction (CR) and physical exercise have been shown to increase lifespan and reduce cellular senescence [201].

CR, which refers to reducing calorie intake to a level that does not compromise overall health, has been widely considered one of the most promising dietary interventions to extend lifespan. Aiken et al. reported that CR intervention effectively reduced glomerulosclerosis and tubular atrophy before the onset of significant age-related changes [202]. CALERIE trials suggested that 2 years of moderate calorie restriction significantly reduced multiple cardiometabolic risk factors in young, non-obese adults [203]. In addition, short-term CR exhibits a renoprotective effect in experimental cisplatin-induced renal injury via inhibition of apoptotic effects and activation of Sirt1 [204].

Frailty is a state of vulnerability to poor resolution of homoeostasis in the elderly, among which physical exercise has been proven to be effective in preventing frailty [205]. Physical exercise improves biological age in a cohort of middle-aged men via an increase in telomerase or a potential recruitment of cells with longer telomeres into peripheral blood [206]. The Lifestyle Interventions and Independence for Elders (LIFE) study showed that exercise intervention slowed the rate of decline in estimated glomerular filtration rate per cystatin C (eGFRCysC) among community-dwelling sedentary older adults compared with health education [207]. Aerobic exercise can improve CKD-induced muscle wasting by inhibiting the overexpression of inflammatory factors, reducing oxidative stress, and stabilizing mitochondrial function and autophagy [208]. In hypertensive rats, exercise training reduces renal fibrosis by downregulating inflammatory and profibrotic pathways [209]. Collectively, calorie restriction and physical exercise play a crucial role in mitigating kidney aging and CKD.

## 7. Conclusions and Perspectives

Accumulating evidence has demonstrated that the process of kidney aging undergoes structural and functional alterations similar to those observed in CKD. Senescence and the associated SASP have been identified as the primary drivers leading to structural and functional degeneration in kidney aging and aging related kidney diseases. In the past decade, our understanding of the roles and mechanisms of cellular senescence in aging and CKD has been significantly improved.

**Figure 4:**
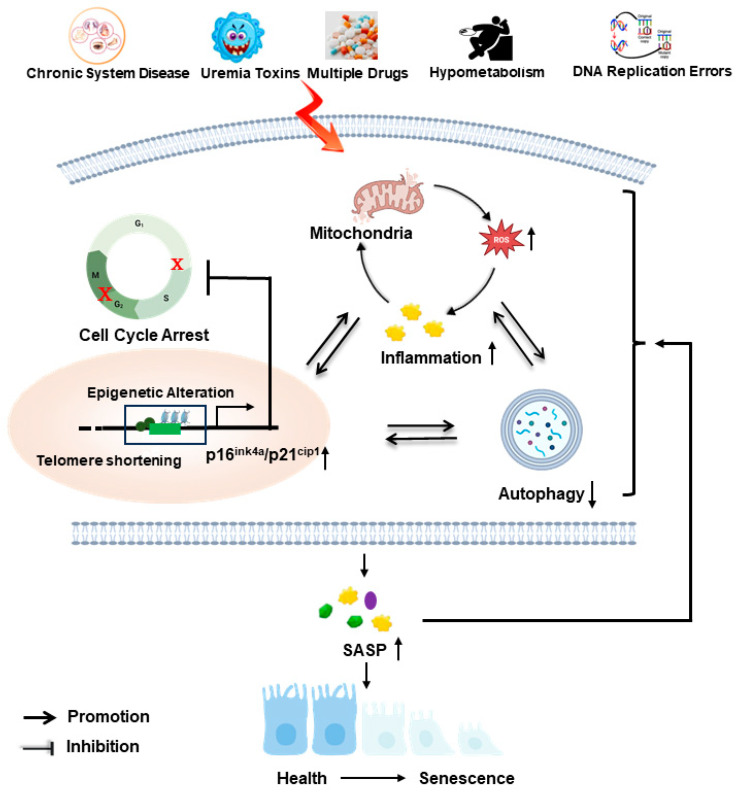
Schematic illustration of a crosstalk network in senescent cells. Continuous damage induced by endogenous and exogenous stimulation, including uremia toxins, hypometabolism, DNA replication errors, chronic ischemia, etc., leads to mitochondrial dysfunction, resulting in ROS release and inflammation activation, which forms a feedback cycle and a decrease in autophagy. Simultaneously, the reduction in autophagy activity fails to eliminate impaired/damaged organelles mediated by the mitochondria-ROS-inflammation cycle. Both the mitochondria-ROS-inflammation cycle and the decreased autophagy induce epigenetic alterations that regulate telomere shortening and the transcription of senescence-related genes such as p16 and p21, leading to cell cycle arrest and the release of SASP. The sustained release of SASP exacerbates mitochondrial damage, further decreases autophagy activity, and increases epigenetic changes, resulting in the formation of a detrimental feedback loop to enhance cellular senescence; ↑: upregulation; ↓: downregulation.

It is important to note that a regulatory network, including mitochondrial damage, oxidative stress, the decrease in autophagy, and the alteration in epigenetic regulation triggered by endogenous and exogenous stimulation, dynamically governs cellular senescence and the release of SASP [210]. Cellular senescence and the released SASP can further deteriorate mitochondrial dysfunction, subsequently leading to an increase in the production of ROS, the activation of inflammation, and the reduction in autophagy, which forms a detrimental feedback network (Figure 4). Thus, the regulation of senescence is a highly dynamic and complicated process, and there are numerous unresolved questions and knowledge gaps requiring further investigation. The utilization of cutting-edge technologies such as single-cell RNA sequencing, spatial transcriptomics, multiplex immunohistochemistry, and relevant bioinformatics analyses should facilitate exploring novel characteristics and intricate regulation of cellular senescence, defining the specific molecular targets of senescent cells, and uncovering the heterogeneity and dynamics exhibited by renal resident cells during the aging process. Enhanced comprehension of the pathogenic mechanisms underlying senescent cells during aging and CKD will ultimately unveil innovative and efficacious targets for therapeutic strategies against CKD and age-related kidney diseases.

## Figures and Tables

**Table 1:** Therapeutic intervention in aging kidneys.

	Targets	Agents/Compounds	Pharmacological Mechanism	Animal Models
Senostatics	Reducing the generation of SASP	Rapamycin	Inhibition mTOR	24-month-old mice
		Rapamycin analogue (RAD001)	Inhibition mTORC1	20–22-month-old rat
		Metformin	Activation of AMPKInhibition of mTORC1	Senescence-accelerated mouse prone 8-, 24-month-old mouse
		Kaempferol	Inhibition MAPK and NF-ҡB	20-month-old rat
		Resveratrol	Inhibition NF-ҡB	D-gal-induced mouse
Senolytics	Targeting senescent cells	Dasatinib and Quercetin	Inhibition PI3K and BCL2	INK-ATTAC mice
		FOXO4 DRI	Inhibition of FOXO4 and p53	20–24-month-old mice
		ABT-263, ABT-737, ABT-199	Inhibition BCL-2	7 Gy irradiation + bilateral IRI
		PPARα agonist	Activation of AMPK and SIRT1 signaling	18-month-old mice
Cell therapy	HSCs	Bone marrow	Direct parenchymal reconstitution by stem cells	Radiated 12-month-old mice
		KIM-MSC	Kidney targeting to the stenotic kidney and augment homing of MSC coated with an anti-KIM1 antibody to the injured kidney	INK-ATTAC mice + murine renal artery stenosis
Non-pharmaceutical therapy	Nutrient regulators	Calorie restriction	-	24-month-old rat
		Exercise	-	24-month-old mice

SASP: senescence-associated secretory phenotype; mTOR: mammalian target of rapamycin; mTORC1: mechanistic target of rapamycin complex 1; AMPK: AMP-activated protein kinase; NF-ҡB: nuclear factor kappa B; PI3K: phosphoinositide 3-kinase; BCL2: B-cell leukemia/lymphoma 2; FOXO4: fork head box O; PPARα: peroxisome proliferator-activated receptor α; KIM: kidney injury molecule; MSC: mesenchymal stem cell.

## Data Availability

Not applicable.
